# SP70-Targeted Imaging for the Early Detection of Lung Adenocarcinoma

**DOI:** 10.1038/s41598-020-59439-9

**Published:** 2020-02-13

**Authors:** Jian Xu, Shichang Zhang, Wei Zhang, Erfu Xie, Min Gu, Yue Wang, Lu Yang, Bingfeng Zhang, Jiexin Zhang, Chunrong Gu, Ting Xu, Daqian Li, Fang Wang, Peijun Huang, Shiyang Pan

**Affiliations:** Department of Laboratory Medicine, The First Affiliated Hospital of Nanjing Medical University (Jiangsu Province Hospital), Nanjing, China

**Keywords:** Cancer imaging, Non-small-cell lung cancer

## Abstract

NJ001 is a monoclonal antibody that can specifically recognize the SP70 antigen on lung adenocarcinoma cells. The goal of this study was to explore its utility in targeted imaging. Subcutaneous xenograft and orthotopic lung tumor implantation BALB/c mouse models were established. Near-infrared fluorescent CF750-labeled NJ001 was injected into two tumor mouse models. Mice that received orthotopic lung tumor implantation were also injected with NJ001-conjugated nanomagnetic beads intravenously, and then underwent micro-CT scanning. Meanwhile, mice with lung tumor were intravenously injected with normal saline and bare nanomagnetic beads as a control. Fluorescence could be monitored in the mice detected by anti-SP70 fluorescence imaging, which was consistent with tumor burden. Signal intensities detected with SP70-targeted micro-CT scans were greater than those in control mice. More importantly, orthotopic tumor lesions could be found on the fourth week with SP70-targeted imaging, which was 2 weeks earlier than detection in the control. Our results suggest that SP70 is a promising target for molecular imaging, and molecularly targeted imaging with an NJ001-labeled probe could be applied for the early detection of lung adenocarcinoma.

## Introduction

Lung cancer is the leading cause of cancer-related death in humans^1,2^. Approximately 1,590,000 people died from lung cancer in 2012^3^. Five-year survival rates vary from 4–17%, depending on stage and regional differences^4^. Late diagnosis makes treatment options challenging, but early detection could significantly reduce mortality in lung cancer patients^5–7^.

Different imaging modalities can be used for lung cancer detection, such as computed tomography (CT), magnetic resonance imaging (MRI), and ^18^F-fluorodeoxyglucose positron emission tomography and computed tomography (FDG-PET/CT). However, they lack sufficient specificity for the early detection of tumors^8^. Molecularly targeted tumor imaging with a specific monoclonal antibody is superior to conventional nonspecific imaging technologies^9,10^. Nevertheless, there are few reports on molecularly targeted imaging for the detection of lung cancer, because of the lack of molecular targets with high specificity for lung cancer.

SP70 protein is a novel tumor marker of non-small-cell lung cancer (NSCLC), especially lung adenocarcinoma^11^. SP70’s monoclonal antibody NJ001 could specifically recognize and react to lung adenocarcinoma cells^12^. In this study, we aimed to demonstrate the feasibility of SP70-targeted imaging with NJ001-conjugated nanomagnetic beads (immuno-nanomagnetic beads) in lung adenocarcinoma and the potential for its use in real-time monitoring and early detection.

## Results

### Bioluminescence intensity correlated with cell number

Both SPC-A1-luc and U87-luc cells were diluted by a serial 2-fold dilution method, and then luciferase activity was measured. Bioluminescence intensity was highly correlated with the total number of cells (*R*^2^ > 0.9900) for both cell lines (Fig. S1). Furthermore, the average luciferase activity value of SPC-A1-luc *in vitro* was 1,223 photons/sec/cell.

### SP70-targeted fluorescence imaging in subcutaneous xenograft mouse models

**Figure 1:**
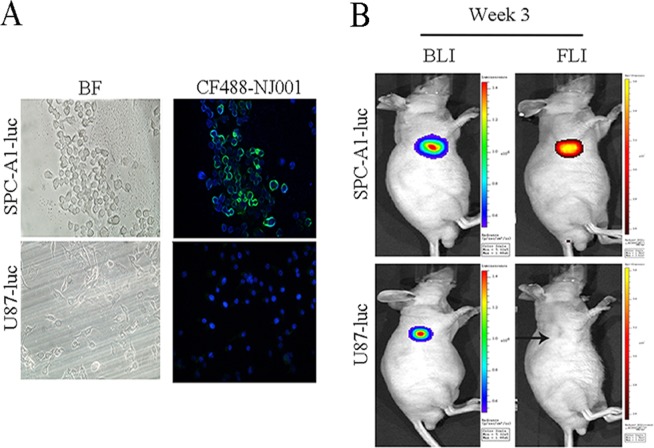
SP70-targeted fluorescence imaging in subcutaneous xenograft mouse models. **(A**) SP70 was located on the SPC-A1-luc cell membrane and in the cytoplasm, but was not expressed in U87-luc cells. (**B**) SPC-A1-luc subcutaneous xenograft tumor could be detected by fluorescence imaging using NIR fluorescence CF750 (red)-labeled NJ001 3 weeks after inoculation. In contrast, U87-luc xenograft tumors could not be detected by fluorescence imaging (black arrow).

DIF showed that SP70 was located on the SPC-A1-luc cell membrane and in the cytoplasm but was not expressed in U87-luc cells (Fig. 1A). After subcutaneous implantation, three mice bearing subcutaneous SPC-A1-luc cell tumors were monitored weekly by both BLI and SP70-targeted fluorescence imaging in parallel. Three mice injected with U87-luc cells were used as controls. For SP70-targeted fluorescence imaging, fluorescent signals could be detected in the subcutaneous SPC-A1-luc mice starting in the third week. However, there were no fluorescent signals in the mice bearing glioma U87-luc cells (Fig. 1B). BLI could detect SPC-A1-luc xenograft tumor development by day 7, and bioluminescence intensity increased in parallel with tumor volume measured by calipers.

### SP70-targeted fluorescence imaging in orthotopic lung tumor models

**Figure 2:**
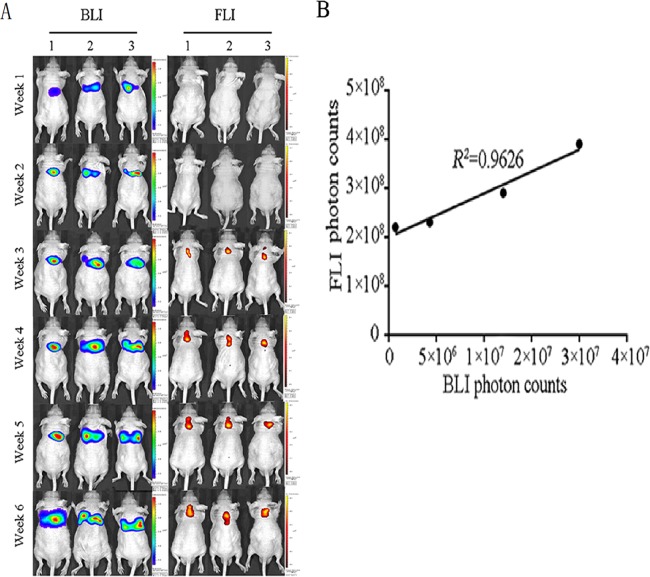
Tumor monitoring with SP70-targeted fluorescence imaging in orthotopic lung tumor implantation models. **(A**) Three mice with SPC-A1-luc orthotopic xenograft tumors were imaged by both BLI (left) and fluorescence imaging (right) at the 3^rd^, 6^th^ and 9^th^ weeks. (**B**) FLI photon counts were correlated with BLI photon counts.

Three mice with SPC-A1-luc orthotopic xenograft tumors were imaged with both BLI and fluorescence imaging. As shown in Fig. 2, SP70-targeted fluorescence imaging could detect the lesions at the third week. Meanwhile, the total lung BLI photon count was 7.2 × 10^5^ photons/sec. Since *in vitro* BLI analysis showed that the luciferase activity value of a single SPC-A1-luc cell was 1,223 photons/sec, it was inferred that the tumor cell number reached approximately 600 on the third week.

### Immuno-nanomagnetic bead characterization

**Figure 3:**
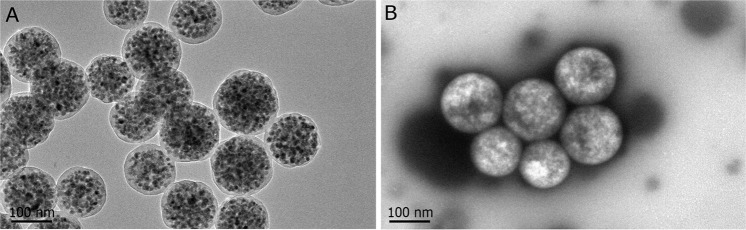
Immuno-nanomagnetic beads characterization with TEM. (**A**) TEM image of the nanomagnetic beads, showing a diameter of approximately 180 nm; (**B**) TEM image of NJ001-conjugated nanomagnetic beads, using phosphotungstic acid for background staining.

NJ001 antibody-coated and noncoated nanomagnetic beads were characterized using transmission electron micrograph (TEM) with phosphotungstic acid staining (Fig. 3).

### Signal enhancement of SP70-targeted micro-CT scan in orthotopic lung tumor models

**Figure 4:**
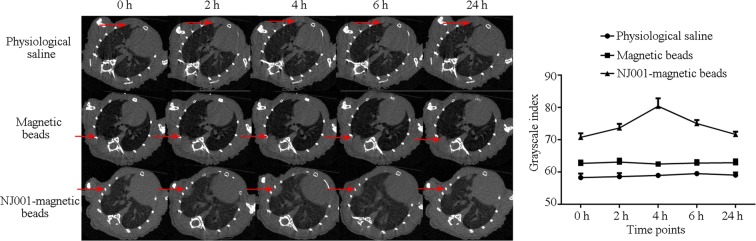
Signal enhancement in SP70-targeted micro-CT scan in orthotopic lung tumor implantation models. Micro-CT scan at 0, 2, 4, 6 and 24 h after NJ001 conjugated nanomagnetic beads or control injection in the sixth week. The image density increased and peaked at 4 h postinjection with NJ001-conjugated nanomagnetic beads.

After injection of the immuno-nanomagnetic beads, the micro-CT signal intensity of the orthotopic lung tumors increased significantly. The image density peaked at 4 h after injection of the immuno-nanomagnetic beads. The tumor grayscale index in the immuno-nanomagnetic beads group was the highest among the three groups (Fig. 4).

**Figure 5:**
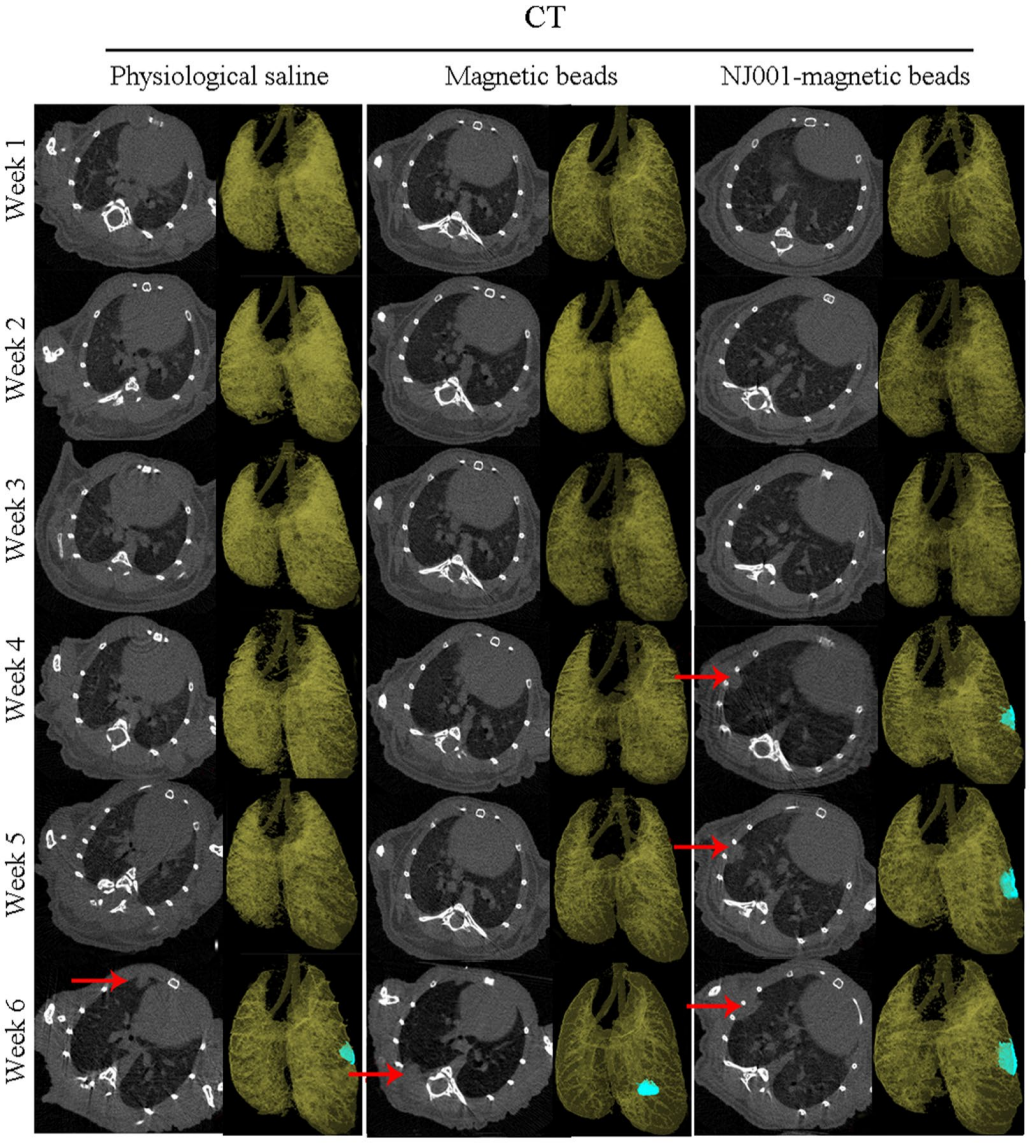
Earlier tumor detection with SP70-targeted micro-CT imaging. SPC-A1-luc orthotopic xenograft tumors were detected by micro-CT scan weekly. Each group contained three mice. One image of each group is shown.

Tumor lesions could be detected at the sixth week in the mice injected with normal saline or bare nanomagnetic beads but were visible by the fourth week in the mice receiving immuno-nanomagnetic beads (Fig. 5). In other words, orthotopic tumor lesions could be found 2 weeks earlier by SP70-targeted micro-CT scan compared with routine micro-CT scan.

## Discussion

The insidious onset of lung adenocarcinoma usually results in metastases at the time of initial diagnosis^13,14^. Early diagnosis of lung adenocarcinoma is challenging. Traditional imaging examinations such as CT and MRI are based on changes in tissue morphology^15–18^, and often lack sufficient specificity to fully predict a tumor’s behavior^19–21^. Therefore, it is essential to find tumor-specific molecules and establish reliable imaging methods for accurate diagnosis.

The SP70 protein is expressed in lung adenocarcinoma but not in normal tissue, blood cells or other cell lines, such as U87 (a glioma cell line). And NJ001 also exhibits anti-tumor activity against NSCLC both *in vitro* and *in vivo*^12^. SP70 is detectable in the serum of patients with lung adenocarcinoma. We also found that SP70 is a key protein that can regulate the expression of numerous genes (GEO accession number: GSE59655), promoting cancer cell proliferation and metastasis. Molecularly targeted imaging will allow clinicians to not only to see where a microscopic tumor is located in the body at an early stage, but also to specifically visualize the biological characteristics of tumors^22–24^. However, biomarker discovery becomes challenging because only a few biomarkers can be both diagnostic and prognostic^25^. Thyroid transcription factor-1 (TTF-1) and napsin A have been well known as lung adenocarcinoma biomarkers for immunohistochemistry^26^. However, they are not suitable for the application of targeted imaging technology due to their nononcogenic origins. Recently, Predina *et al*. reported that folate receptor alpha (FRα)-targeted intraoperative molecular imaging might be used during surgery^27^.

Three kinds of *in vivo* molecular imaging techniques were developed in this study: BLI based on the activity of luciferase that catalyzes the substrate luciferin in transfected cells, targeted fluorescence imaging with CF750-NJ001, and targeted micro-CT with NJ001-conjugated nanomagnetic beads. Since bioluminescence signal intensity positively correlates with tumor volume, we could perform a quantitative assessment of tumor growth *in vivo*. It was reported that molecular fluorescence imaging with near-infrared fluorescence could be used for detecting tumors and guiding surgery^28–30^. We chose the near infrared fluorescent dye CF750 to label NJ001 due to the wavelength’s enhanced tissue penetrability and the dye’s favorable pharmacokinetic properties. The fluorescent signal perfectly matched the bioluminescence signal. Conversely, there were no fluorescent signals in mice bearing glioma U87-luc cells, which were negative for the SP70 antigen. These results demonstrated that CF750-NJ001 had high specificity for lung subcutaneous xenografts.

Furthermore, lesions in orthotopic lung models could be detected by SP70-targeted fluorescence imaging at the third week, while positive signals were found at the sixth week by conventional micro-CT. Considering photon attenuation when penetrating the body cavity, lesions may contain more than 600 tumor cells. Routine CT scans can only detect lesions of more than 1.5 mm lesions which contain approximately10^6^ tumor cells. This result indicates that SP70-targeted fluorescence imaging is much more sensitive than traditional imaging techniques. Therefore, SP70 acts as an oncogenic protein and could be a target of molecular imaging, even for micrometastasis. Because of the weak penetration ability in the human body, SP70-targeted fluorescence imaging might only be used to locate lung adenocarcinomas and identify positive margins during surgery.

CT is the first-line imaging tool for lung cancer diagnosis. Though contrast agents can enhance the resolution of CT, they cannot improve its ability to differentiate malignant from benign tumors. Current contrast agents are mainly (approximately 90%) excreted by kidneys. A larger dose of contrast agent often leads to the occurrence of nephropathy^31^. Nanotechnology could be used in molecularly targeted imaging. Nanoparticles easily penetrate cancerous tissue capillaries^32^, and can be deposited preferentially in cancer tissue. Recently, Patrick *et al*. used CNA35-conjugated gold nanoparticles as a CT contrast agent for the molecular imaging of myocardial scars^33^. In our study, the diameter of the nanomagnetic beads was 180 nm, so they could easily be distributed into cancer tissue. NJ001-conjugated nanomagnetic beads could specifically identified lung adenocarcinoma cells, and molecularly targeted enhancement significantly improved the sensitivity of micro-CT imaging for monitoring tumor growth. Lesions were detected at the fourth week in the SP70-targeted micro-CT group, 2 weeks (one third of the amount of time) earlier than in the groups injected with normal saline and bare nanomagnetic beads controls. It could be inferred that SP70-targeted imaging could greatly shorten the period from tumor onset to diagnosis, and therefore accomplish the aim of detecting the early stage of lung adenocarcinoma. Although the antigenicity of the murine monoclonal antibody NJ001 was minimized with conjugation to the nanomagnetic beads, its biosafety and metabolic properties remain to be determined.

In conclusion, SP70-targeted imaging can markedly improve the detection of lung adenocarcinoma detection. Molecularly targeted imaging with NJ001-labeled probes may have precision medical applications for the early diagnosis of lung adenocarcinoma.

## Materials and Methods

### Materials

Superparamagnetic polymer nanospheres were purchased from Shanghai Allrun Nano New Science & Technology Ltd. (Shanghai, China). Bovine serum albumin (BSA), ethylcarbodiimide (EDC) and N-hydroxysuccinimide (NHS) were purchased from Sigma-Aldrich (St. Louis, MO, USA). The CF488 Dye Antibody Labeling Kit and the CF750 Dye Antibody Labeling Kit, which are both fluorescent succinyl ester dye kits, were purchased from Biotium (Hayward, CA, USA). D-luciferin was purchased from Fanbo Biochemicals (Beijing, China).

### Cell culture

SPC-A1-luc cells (SP70-positive) and U87-luc cells (SP70-negative), cell lines derived from human lung adenocarcinoma and human glioma, respectively, that express luciferase by stable transfection with the firefly luciferase gene, were purchased (Shanghai Baidaian Company, Shanghai, China). Both cell lines were cultured in Roswell Park Memorial Institute (RPMI) 1640 medium containing 50 U/mL penicillin, 50 U/mL streptomycin, and 10% fetal bovine serum (Invitrogen, Carlsbad CA, USA). Cells were grown in an incubator with 5% CO_2_ at 37 °C.

### Animal models

Four-week-old male BALB/c nude mice (Shanghai SLAC Laboratory Animal Co. Ltd. Shanghai, China) were maintained in a pathogen-free environment. All procedures were conducted in accordance with the Animal Care and Use Committee guidelines of Nanjing Medical University. To establish subcutaneous xenograft mouse models, SPC-A1-luc and control U87-luc cells were harvested in the logarithmic growth phase, counted, and resuspended in phosphate-buffered saline (PBS) to a final density of 5 × 10^7^ cells/mL. Next, 5 × 10^6^ SPC-A1-luc cells or U87-luc cells suspended in 0.1 mL of sterile PBS were implanted subcutaneously into the right flank of each mouse. For an orthotopic lung tumor implantation mouse model, 5 × 10^6^ SPC-A1-luc cells suspended in 0.1 mL of sterile PBS were injected into mice intravenously via the tail vein. All animal studies were performed with the approval of the Animal Care and Use Committee of Nanjing Medical University.

### NJ001-conjugated nanomagnetic beads (immuno-nanomagnetic beads)

Superparamagnetic polymer nanospheres with diameters of 180 nm were conjugated with NJ001 antibody. First, 2 mg nanomagnetic beads were activated with 200 µL of 5 mg/mL EDC and NHS in a 37 °C water bath for 30 min, followed by three washes with phosphate-buffered saline with tween 20 (PBST). Second, the beads were mixed with 50 µL of 5 mg/mL NJ001 antibody and again incubated in a 37 °C water bath for 4 h. Third, after three more washes with PBST, the beads were resuspended in 1 mL of PBST containing 5% BSA and gently shaken at 4 °C overnight. Finally, the supernatant was removed, and the beads were resuspended in 200 µL of PBST and kept at 4 °C.

### Immuno-nanomagnetic bead characterization

TEM were taken by using a JEM-2100 HR transmission electron microscope (JEOL, Tokyo, Japan). The nanomagnetic bead sample (5 µL) was dropped onto copper 200-mesh grids that were pretreated with UV light to reduce static electricity. After 30 min, the solvent was drained with filter paper, and a phosphotungstic acid stain solution (1% by weight, adjusted to pH 6.0) was applied for 30 s. After drying, TEM imaging was performed.

### Direct immunofluorescence (DIF) analysis

The Dye Antibody Labeling Kit was used to develop the fluorescently dyed probe CF488-NJ001 for *in vitro* DIF. SPC-A1-luc and U87-luc cells were grown on coverslips, fixed in 95% ethanol for 15 min at room temperature and blocked with 5% BSA for 30 min at 37 °C. After washing three times with PBS, the coverslips were incubated with CF488-NJ001 (1:200 dilution) for 30 min at 37 °C. After three washes, a fluorescent dye, 4′,6-diamidino-2-phenylindole (DAPI) was subsequently added for nuclear staining. Samples were examined using an Olympus IX71 inverted fluorescence microscope (Olympus Optical Co. Ltd, Tokyo, Japan) coupled with a charge-coupled device (CCD) camera (ProgRes, Jenoptik/Jena, Germany).

### Bioluminescence imaging (BLI)

Mice were injected intraperitoneally with 150 mg/kg of D-luciferin in PBS, and imaged with IVIS 2000 imaging system (Caliper Life Sciences, Hopkinton MA, USA). Analyses of the BLI images were performed using Living Image software from Caliper Life Sciences by drawing regions of interest over the tumor region and obtaining maximum values in photons per second per cm^2^ per steradian or total flux as photons per second. BLI was repeated weekly.

### Fluorescence imaging (FLI)

Fluorescence imaging was performed using the CF750 Dye Antibody Labeling Kit, a fluorescent succinyl ester dye kit, to label NJ001 for *in vivo* fluorescence imaging analysis. A dose of 5 µg of CF750-NJ001 in 100 µL PBS was injected via the tail vein one week after the cell inoculation and weekly thereafter. The fluorescence intensity in each mouse was assessed 48 h after injection using the same IVIS 2000 imaging system with an excitation wavelength of 755 nm and an emission wavelength of 800 nm. Fluorescence from the ROI was defined manually, and fluorescence efficiency was expressed as (photons/s) ÷ (µW/cm^2^)^34^. Images were analyzed using Living Image software.

### Micro-computed tomography (micro-CT)

Orthotopic mouse models were imaged with BLI to monitor tumor growth one week after SPC-A1-luc cell inoculation. Nine mice with no significant difference in tumor size were selected for the following micro-CT scans. Three orthotopic mice were injected intravenously with 100 µL NJ001-conjugated nanomagnetic beads, three mice were injected with 100 µL normal saline and three mice were injected with bare nanomagnetic beads as controls. Micro-CT scans were acquired at 0, 2, 4, 6 and 24 h after injection to determine tumor lesion and size. Micro-CT was performed on a SkyScan 1176 (SkyScan NV, Kontich, Belgium), a small animal imager, at 50 kV and 490 µA. A total of 360° views were acquired at 1° angle increments, each for an exposure time of 120 ms, to give a resolution of 35 µm using external respiratory gating. SkyScan software was used for multiplanar and 3D image reconstruction.

### Statistical analysis

Statistical analysis was conducted using SPSS Statistics v20.0 (SPSS Inc., Chicago IL, USA) and GraphPad Prism v6.0 (GraphPad Software, San Diego CA, USA). Dunnett’s test was used to compare the experimental group to the control groups undergoing micro-CT scans. The correlation was analyzed by the Pearson correlation coefficient. All statistical assessments were performed in a two-sided setting, using a significance cut-off of 0.05.

## Supplementary information

Supplementary information

## Data Availability

All data generated or analysed during this study are included in this published article (and its Supplementary Information Files).
