# Supplementary material for: SP70-Targeted Imaging for the Early Detection of Lung Adenocarcinoma
Source: Sci Rep. 2020 Feb 13;10:2509. doi: 10.1038/s41598-020-59439-9 (PMC7018733; doi:10.1038/s41598-020-59439-9)
Supplement: Supplementary file 1 — Supplementary information [file 41598_2020_59439_MOESM1_ESM.pdf]

SP70-Targeted Imaging for the Early Detection of Lung Adenocarcinoma

Jian Xu[#], Shichang Zhang[#], Wei Zhang, Erfu Xie, Min Gu, Yue Wang, Lu Yang, Bingfeng Zhang, Jiexin Zhang, Chunrong Gu, Ting Xu, Daqian Li, Fang Wang, Peijun Huang, Shiyang Pan[*]

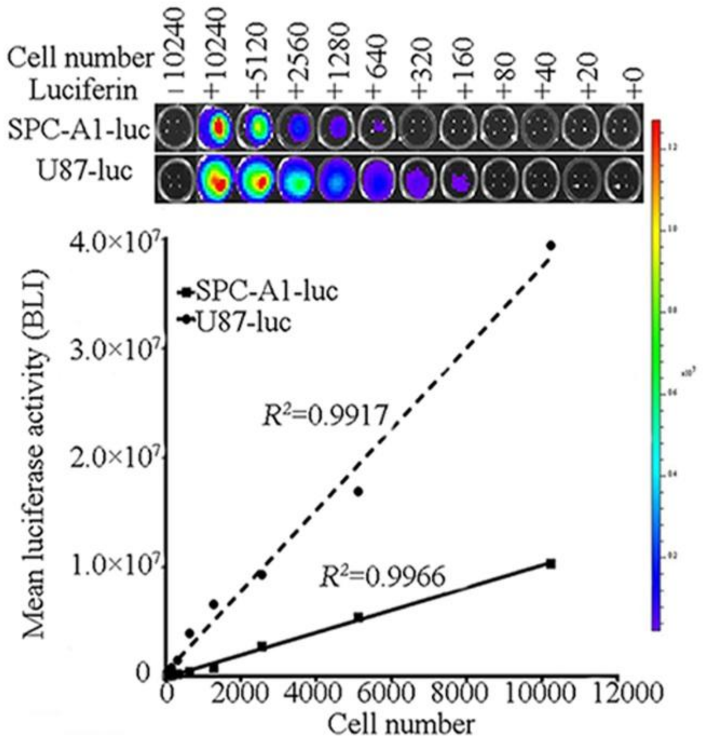

Figure S1. Luciferase activity correlated with tumor cell quantity. Serially diluted SPC-A1-luc and U87-luc cells from 20 to 10240 cells were cultured in 96-well plates, leaving one empty well. 1 μL D-luciferin (1 μL, 15 mg/mL) was added to each well, except for one well. The wells without the addition of luciferase substrate or with no cells served as the blank control and negative control, respectively. After 10 min of incubation at room temperature, the plates were scanned.
